# Iberogast®-Induced Acute Liver Injury—A Case Report

**DOI:** 10.1016/j.gastha.2022.02.020

**Published:** 2022-05-19

**Authors:** Alice Leroy, Henri Perrin, Raphael Porret, Christine Sempoux, Haithem Chtioui, Montserrat Fraga, Pierre-Alexandre Bart

**Affiliations:** 1 Service de Médecine interne, CHUV, Lausanne, Switzerland; 2 Service de Pathologie Clinique, CHUV, Lausanne, Switzerland; 3 Service de Pharmacologie clinique, CHUV, Lausanne, Switzerland; 4 Service de Gastro-entérologie et d’hépatologie, CHUV, Lausanne, Switzerland

**Keywords:** Iberogast®/STW 5, Greater Celandine, Drug-Induced Liver Injury (DILI), Herbal Preparations, Phytotherapy

## Abstract

Alternative medicines such as phytotherapy and herbal preparations have been widely used over the past 5 decades. However, they are still poorly known in Western medicine, and because they are considered as natural products, they are often omitted in the review of medication. One of the most used herbal preparations in Europe is Iberogast®, a formulation of 9 medicinal plant extracts, including Greater Celandine that has proven effective in the treatment of functional dyspepsia and irritable bowel syndrome. Safety and tolerability of Iberogast® were extensively evaluated in double-blind and randomized studies vs placebo, but rare and usually mild adverse symptoms have been reported in the literature. We report a 32-year-old female with no previous medical history who presented to the emergency department with abdominal pain, jaundice, and pruritus. The blood tests revealed an acute severe hepatitis with marked increase of direct bilirubin. After exclusion of other possible acute liver injury etiologies, we retained the diagnosis of Iberogast®-associated drug-induced liver injury. Patient’s symptoms resolved spontaneously 5 weeks after treatment interruption. Despite the general safety of Iberogast®, occasional cases of drug-induced liver injury have been documented. Based on these observations, we recommend that the use of herbal and phytotherapeutic products should be part of the standard investigation of the medical history, as they could be relevant information in the diagnosis process of acute liver injury.

## Introduction

Herbal preparations and dietary supplements (HDS) have become more popular in the last decades. They are perceived as generally safe, but their broad range of uses as over-the-counter alternative treatment is also associated with nonnegligible adverse events. A Spanish study showed that HDS were responsible for drug-induced liver injury (DILI) as often as isoniazid with more than a half of the cases requiring hospitalization.^1^ Iberogast® is a phytotherapeutic drug containing extracts of 9 different herbs (*Iberis amara*, *Chelidonii herba*, *Matricariae flos*, *Melissae flos*, *Liquiritae radix*, *Menthae piperitae folium*, *Cardui mariae fructus*, *Carvi fructis*, and *Angelicae radix*). It is one of the most widely used phytotherapeutic drugs in the world, being sold for more than 50 years with dozens of millions of users.^2^ It has been proved more effective than placebo in the treatment of functional dyspepsia and irritable bowel disease.^2^^,^^3^ In addition to its effectiveness, the drug is also commonly considered to be very safe, with a rather low rate of adverse effects, most of which being mild to moderate.^4^^,^^5^ Nevertheless, occasional episodes of DILI attributed to Iberogast® components and mostly to Greater Celandine (*Chelidonium majus*) have been reported in the scientific literature, with 1 case of acute liver failure requiring liver transplantation.^6^

## Case Report

A 32-year-old healthy Caucasian female presented to the emergency department for a 3-day history of progressive abdominal pain, jaundice, and pruritus. In the previous year, the patient had suffered from multiple episodes of abdominal discomfort attributed to irritable bowel syndrome without jaundice and/or pruritus, which always resolved spontaneously. These abdominal pain episodes were often associated with constipation and occasional diarrhea. The patient started a treatment of Iberogast® 6 weeks before admission. Her only other medical treatment was a progestin-only pill, started 14 years ago, and occasional use of paracetamol, never more than 3 g/d. She reported eating once edible mushrooms she had bought in a regular store days before the event. There was no history of drug use, alcohol abuse, or promiscuous sexual behavior. The patient’s unique comorbidity was an intolerance to histamine diagnosed 6 years before.

Physical examination revealed a generalized jaundice and a painful abdominal palpation, especially in the right upper quadrant. Bowel sounds were normal, and there were no hepatomegaly, splenomegaly, masses, or peritonism.

Liver function tests at admission showed a marked cytolysis associated with hyperbilirubinemic cholestasis: aspartate aminotransferase 235 U/L (upper limit of norm [ULN] 32 U/L), alanine transaminase (ALT) 469 U/L (ULN 36 U/L), bilirubin 150 μmol/L (ULN 21 μmol/L) with conjugated bilirubin 100 μmol/L, phosphatase alcaline 134 U/L (ULN 108 U/L), and gamma-glutamyl transferase 156 U/L (ULN 42 U/L). With a ratio (R) ALT/AP of 10, the pattern of liver damage was hepatocellular, according to the International Consensus Meeting criteria. Complete blood count was normal and did not show eosinophilia; electrolytes and kidney function were within the normal range, as well as inflammatory parameters. Synthetic liver function was preserved with normal factor V. Urine pregnancy test was negative. Other causes of liver injury were ruled out: serologic tests for viral hepatitis A, B, C, and E were negative, and hepatitis E virus RNA levels by real-time polymerase chain reaction assay remained undetectable as well. Serologic tests that detect cytomegalovirus and Epstein-Barr virus antibodies were both positive for immunoglobulin G and negative for immunoglobulin M, which indicated past infections. HIV serology also was negative. Immunologic assessment with electrophoresis and autoimmune markers were normal. Wilson disease was ruled out, in the same way as alpha-1 antitrypsin deficiency and autoimmune liver disease (test for antinuclear, antimitochondrial, antismooth muscle, and anti-LKM-1 antibodies).

An abdominal ultrasound examination was performed and showed a normal anatomy and echogenicity of the liver without any suspect lesion. The portal vein system as well as hepatic veins were permeable. There was no intra- or extra-hepatic bile ducts dilatation. A cholangio-magnetic resonance imaging revealed a suspicion of distal common bile duct stenosis. However, a complementary endoscopic ultrasound excluded any sign of biliary obstruction and found an inflammation of the bile ducts. Ampullary biopsies confirmed some congestion with acute inflammation, without signs of dysplasia or malignancy.

During hospitalization, the patient showed a progressive decrease of the total bilirubin values but a persistent transaminitis (ALT > 15× ULN). We performed a liver biopsy, which showed a pattern of acute lobular hepatitis, with spotty necrosis and numerous foci of histiocytic inflammation (Figure). There was no fibrosis and no significant portal inflammation. Bile ducts were present in all portal tracts without any alteration. Importantly, hepatic copper concentration was calculated to be 34 μg/g (ULN 35 μg/g).

**Figure:**
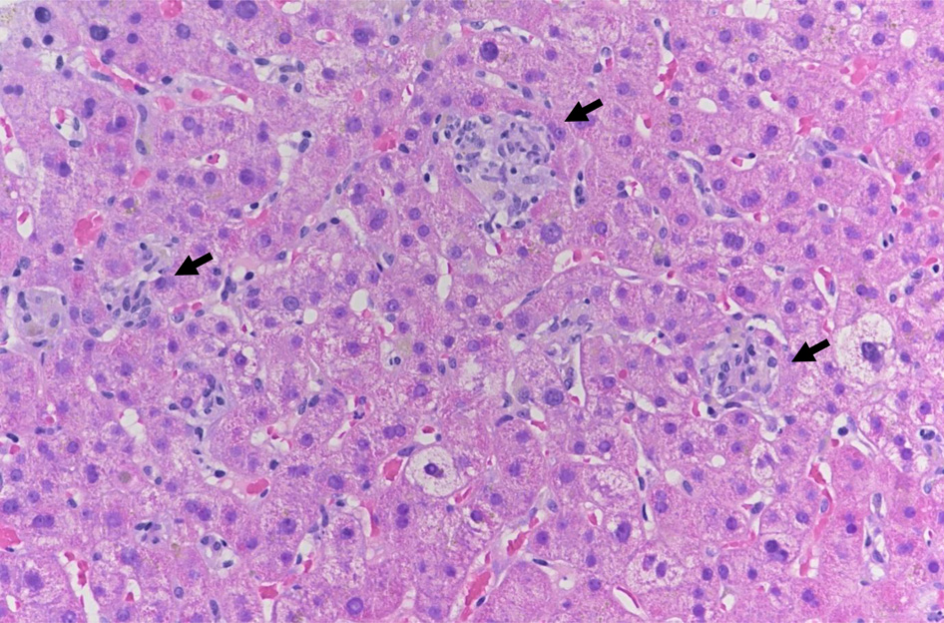
Liver biopsy showing features of lobular hepatitis with multiple foci of histiocytic inflammation (arrows, hematoxylin-eosin staining, original magnification ×200).

This patient presented a symptomatic acute severe hepatitis. Possible infectious, autoimmune, neoplastic, and lithiasic causes as well as a Wilson’s disease were rationally excluded. Thus, as an exclusive diagnosis, we retained Iberogast® responsible for this patient’s clinical presentation. To support our diagnosis, we calculated a Council for International Organizations of Medical Sciences/Roussel Uclaf Causality Assessment Method score, which is widely used in assessing the causality of DILI. The score was at 8 points, which considers our diagnosis as “probable”.^7^

Twenty-eight days after discontinuation of Iberogast®, laboratory results showed a bilirubin concentration of 29 μmol/L, aspartate aminotransferase 25 U/L, and ALT 62 U/L, with a reduction of the ALT of about 90% compared with the peak of 574 U/L.

## Discussion

Iberogast® is a preparation containing several plant extracts. Most of them are not documented for specific hepatotoxicity. One of the main components of Iberogast® is the Greater Celandine, a plant of the Poppy family typically used in the traditional Chinese medicine to treat gastrointestinal discomfort and dyspepsia. Its extracts contain more than 20 ingredients, including multiple biologically active isoquinoline alkaloids, such as benzophenanthridine, protoberberines, and hydroxycinnamic acid derivatives.^7^^,^^8^ Despite its general good tolerance, Greater Celandine is recognized as a possible provider of a typical herb-induced liver injury, with dozens of documented cases. In the majority of these episodes, the patient presents a jaundice associated with a moderate to marked elevation of aminotransaminases levels. In rare instances, severe liver injury was reported. The physiopathogenic mechanism responsible for the liver injury is not precisely known, but the hepatotoxicity features are mostly compatible with an idiosyncratic reaction (most authors supporting the hypothesis of metabolic idiosyncrasy).

Idiosyncratic DILI is an unpredictable dose-independent reaction. Actually, studies suggest a complex combination of genetic predisposition, nongenetic susceptibility (age, female sex, drug interactions, and cross-sensitization), and exposure to environmental factors.^9^ Regarding genetic risk factors, the data are still relatively limited, but there has been growing interest in multiple HLA polymorphisms genes as well as genes involved in drug metabolism and hepatocellular transport systems.^10^

Indeed, there is increasing evidence that genetically determined functional impairment of bile acids transporters plays an important pathophysiological role in the development of DILI.^11^, ^12^, ^13^ We, therefore, proceeded, with our patient’s consent, to blood sampling for DNA extraction. We specifically examined the genes encoding for the hepatic ducts’ bile acids transporters (ABCB11), phospholipids (ABCB4), and aminophospholipides (ATP8B1) as well as the Farsenoid-X-receptor (NR1H4). Mutations and polymorphisms in these genes have been proposed to increase the risk of DILI.^11^ Interestingly, we found the presence of a homozygous polymorphism in the ABCB11 gene (A p.A444V) as well as a second homozygous variant c.711A>T in the ABCB4 gene. Importantly, the homozygous polymorphism at position 444 in ABCB11 gene was already previously associated with DILI.^11^ Moreover, drugs inhibiting ABCB11 are also associated with the possible occurrence of drug-induced cholestasis. Here, the genetic underlying background may have played a role in the profound cholestatic DILI picture after Iberogast® exposure.

It is important to keep in mind though that genes involved in the bile salt export pump are highly redundant and polymorphic. Mutations in these genes are common in the general population and may only constitute a general susceptibility to DILI. Further research is needed to determine genetic risk factors for specific drugs or populations to guide treatment decisions (personalized medicine).

Despite the general good tolerance of Iberogast®, prescribers should be aware of the potential side effects of this drug and discontinue the treatment in case of compatible symptoms or biological affectations. Besides, recurrence after inadvertent readministration has been documented,^4^ and patients with a history of DILI should avoid reexposure to this drug but also to any other HDS potentially containing Greater Celandine. Globally, this case report is also a reminder that herbal preparations contain active ingredients that can be responsible for severe adverse effects and should therefore always be investigated in the drug history. According to the United States DILI Network, which collects data on patients with DILI since 2004, DILI cases caused by HDS are increasing.^14^ One reason is that HDS are not subject to the same regulations as prescription medicines.

DILI and herb-induced liver injury diagnoses remain challenging, especially when associated with HDS. This might have many explanations. First, clinicians are not used to manage alternative medicines, and very little emphasis is made during their training regarding this issue. Second, as frequently considered as harmless natural products by their consumers, patients might not declare their herbal preparation use to their physician. Moreover, each plant extract may contain several substances that are difficult to standardize, and additional contamination may also occur. Finally, no validated biomarkers exist, and the diagnosis is mostly exclusive. Therefore, efforts should be made to investigate the use of herbal and phytotherapeutic products in a medical history, especially in case of atypical clinical presentations, as recommended by the European Association for the Study of the Liver.^15^
